# Bacteriology and Disease

**Published:** 1906-02-03

**Authors:** 


### Bacteriology and Disease.

The recent announcement by Drs. Ford Robertson and McRae of their discovery of a new bacillus in the blood and cerebro-spinal fluid of patients suffering from general paralysis is likely to give a new and much-needed impulse to the scientific study of this and other forms of insanity. It is, of course, in the highest degree probable that all these forms have their origin in some depravation of the fluids bathing the nervous structure and derived immediately from the blood; and the histories of alcoholic or drug intoxication, with the perversion of intellectual faculty which attend upon the subjects of them from the first, and the structural degenerations which supervene when they have been long continued, have presented analogies to insanity which it has long been impossible to overlook. The first suggestion from these analogies, however, would probably be in the direction of toxins created by defective metabolism rather than by bacillary growth; although there is manifestly no reason why the latter should not fulfil all the conditions of the problem. The evidence of its doing so cannot be regarded as complete until the four tests laid down by Koch have all held good in a sufficient number of instances. These are: (1) that the bacillus must be present in every case of the disease, and not in cases of other diseases; (2) it must be isolated and grown on artificial media; (3) its cultures injected into an animal body must produce the disease in question; (4) the bacillus must again be found in the body in which the disease was thus produced. As regards the applicability of these tests, it fortunately happens that the changes of nutrition incidental to general paralysis, both in the nervous system and in the bones, are almost as characteristic of the disease as either the delusions of exaltation or the impairment of speech, both of the latter being symptoms which would elude investigation in the rodents or other animals likely to be made the subjects of experimental inoculation. For the present, at least, it would be imprudent to build too much upon Drs. Robertson's and McRae's alleged discovery; in the first place, because the mere presence of a previously unrecognised bacillus is no more than a fact requiring further investigation; in the second place, because the results obtained by new inquirers in the vast field of bacteriology must always be regarded as matters requiring confirmation by time and by the work of other observers. Whether this confirmation be obtained or not, the question which has been raised is one of supreme importance to the community. The steady increase of insanity during the last fifty years is one of the most alarming of all adverse conditions of the public health; and the extent to which the subjects of the disease or diseases concerned have been suffered to fall under the control of law and lawyers, instead of under that of doctors, has presented, and still presents, almost insurmountable obstacles in the way of either scientific investigation or of scientific treatment. If the toxins concerned in the production of insanity can once be identified, a great step will have been taken in the direction of reversing the faulty policy of which we have so long been victims, and of restoring the insane to the position and privileges of ordinary citizens.

Another interesting advance in bacteriology has been placed on record by Dr. Klein, F.R.S., in a report furnished to the Local Government Board and issued last week as a Parliamentary paper. Dr. Klein has ascertained that the dried tissues of animals dead of plague, in which the bacilli originally present have been killed by desiccation, nevertheless retain the power of communicating the disease to animals into which they are injected, and which die with all the symptoms of plague, but without themselves containing any bacilli. The same dried tissues, injected in smaller doses, have been found to confer immunity upon the rodents receiving them; and, as the result of a large number of carefully-conducted experiments, Dr. Klein announces that he has succeeded in obtaining a new plague prophylactic of very definite action, which can be prepared in ten or twelve days, which can be easily standardised, and which, being in the form of a dry and sterile powder, can be preserved indefinitely without change. It has been found to confer upon rats and guinea pigs a complete immunity, lasting many weeks, from the effects of injections of virulent bacilli pestis, and we shall hope to hear before long that it has been applied successfully in the case of man, and that some control of the pestilence in India may be expected to follow from its application.

